# Unraveling the Guardian: p53’s Multifaceted Role in the DNA Damage Response and Tumor Treatment Strategies

**DOI:** 10.3390/ijms252312928

**Published:** 2024-12-01

**Authors:** Han Zhang, Jianxiong Xu, Yuxuan Long, Ayitila Maimaitijiang, Zhengding Su, Wenfang Li, Jinyao Li

**Affiliations:** 1 Xinjiang Key Laboratory of Biological Resources and Genetic Engineering, College of Life Science and Technology, Xinjiang University, Urumqi 830017, China; 107552301112@stu.xju.edu.cn (H.Z.); 107552403493@stu.xju.edu.cn (J.X.); 107552401074@stu.xju.edu.cn (Y.L.); 2 School of Pharmaceutical Science, Institute of Materia Medica, Xinjiang University, Urumqi 830017, China; 007649@xju.edu.cn (A.m.); james_su@xju.edu.cn (Z.S.)

**Keywords:** *TP53*, DNA damage response, tumor suppressor, cancer progression, therapeutic strategies

## Abstract

DNA damage can lead to mutations that can alter the function of oncogenes or tumor suppressor genes, thus promoting the development of cancer. p53 plays a multifaceted and complex role in the DNA damage response and cancer progression and is known as the ‘guardian of the gene’. When DNA damage occurs, p53 is activated through a series of post-translational modifications, which stabilize the protein and enhance its function as a transcription factor. It regulates processes including cell cycle checkpoints, DNA repair and apoptosis, thereby preventing the spread of damaged DNA and maintaining genome integrity. On the one hand, p53 can initiate cell cycle arrest and induce cells to enter the G1/S and G2/M checkpoints, preventing cells with damaged DNA from continuing to proliferate and gaining time for DNA repair. At the same time, p53 can promote the activation of DNA repair pathways, including base excision repair, nucleotide excision repair and other repair pathways, to ensure the integrity of genetic material. If the damage is too severe to repair, p53 will trigger the apoptosis process to eliminate potential cancer risks in time. p53 also plays a pivotal role in cancer progression. Mutations in the p53 gene are frequently found in many cancers, and the mutated p53 not only loses its normal tumor suppressor function but may even acquire pro-cancer activity. Therefore, we also discuss therapeutic strategies targeting the p53 pathway, such as the use of small-molecule drugs to restore the function of wild-type p53, the inhibition of negative regulatory factors and synthetic lethality approaches for p53-deficient tumors. This review therefore highlights the important role of p53 in maintaining genomic stability and its potential in therapeutic strategies for cancer.

## 1. Introduction

In the complex process of cellular regulation, the normal operation and stability of cells are essential for the survival of organisms. In living organisms, DNA is continually subjected to damage from both internal and external factors [1]. If DNA damage is not repaired in time, it may cause gene mutations, leading to the incorrect transmission of genetic information, which in turn may cause abnormal cell function or even cancer, thereby changing the function of proto-oncogenes or tumor suppressor genes and ultimately leading to the occurrence of cancer. To counter this threat, cells have evolved a complex DNA damage response (DDR) mechanism, in which the tumor suppressor protein p53 plays a central role [2,3,4]. The p53 protein is often referred to as the ‘guardian of the genome’ and plays a central role in maintaining cell integrity and preventing malignant transformation [5]. Since its discovery in 1979, the p53 protein has been recognized for its key role in coordinating the cell’s response to various stress signals, particularly those caused by DNA damage [5,6]. Encoded by *TP53*, p53 acts primarily as a transcription factor, regulating gene networks involved in cell cycle arrest, DNA repair, apoptosis and senescence. p53’s versatility is reflected in its ability to respond to multiple cellular stresses, including DNA damage, hypoxia and nucleotide deficiency, thereby maintaining genomic stability by regulating the cell cycle, inducing apoptosis or promoting DNA repair [7,8]. In particular, in the DDR, p53 is rapidly activated through a series of complex molecular mechanisms, such as phosphorylation, dephosphorylation and acetylation, and initiates the expression of a series of downstream genes, thereby achieving the precise regulation of cells’ fates [9]. This versatility enables cells to respond appropriately to genomic damage, thereby avoiding the spread of damaged DNA and maintaining genomic stability. In recent years, as research has progressed, p53’s multiple roles in the DDR have gradually been revealed. It not only regulates cell cycle checkpoints to pause cell cycle progression and provide a time window for DNA repair, but also directly participates in DNA repair processes, such as nucleotide excision repair. In addition to its typical role in the DDR, recent studies have revealed that p53 is also involved in the regulation of metabolism, autophagy, stem cell maintenance and the immune response, highlighting its impact on a wide range of biological processes [8,9,10,11,12,13]. Furthermore, the interaction between p53 and the tumor microenvironment highlights its involvement in cancer metastasis and treatment resistance [14,15]. However, the function of p53 is not unchanging. During cancer progression, p53 often undergoes mutations, thereby losing its normal tumor suppressor activity, and, in some cases, mutant p53 can even promote the malignant progression of cancer [8,16,17]. These mutations not only destroy p53’s anti-cancer function, but also give the protein new oncogenic properties, e.g., gain-of-function mutations [18]. The loss of p53’s function disrupts the delicate balance of cell homeostasis, leading to uncontrolled cell proliferation, genomic instability and cancer progression. Therefore, strategies to restore wild-type p53’s function, inhibit mutant p53 or regulate its regulatory pathways are expected to improve the effectiveness of cancer treatment. In recent years, a deeper understanding of the structural dynamics of p53 and its interactions with other molecules has opened up new avenues for therapeutic intervention.

This review aims to provide an in-depth analysis of the protective role of the ‘guardian of the genome’, p53, in DNA damage repair and cancer progression, exploring the complex molecular mechanisms of p53’s activation and the impact of *TP53* mutations on the cell fate, as well as the therapeutic potential of targeting the p53 pathway in oncology. By providing an in-depth analysis of the biological functions of p53, we hope to elucidate its overall position in cellular defense mechanisms and its role in tumorigenesis and development, offering insights for the development of novel therapeutic strategies.

## 2. Structure and Regulation of p53

### 2.1. Structural Features of the p53 Protein

**Figure 1:**
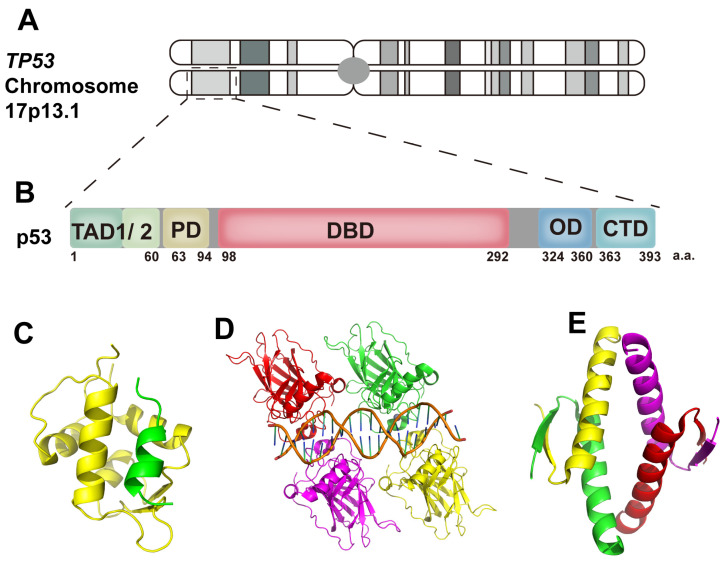
Structure of p53. (**A**) *TP53*, located on chromosome 17p13.1, encodes p53, a protein consisting of 393 amino acids. (**B**) A schematic diagram of the p53 structure [19], which illustrates the following domains: transactivation domain (TAD), proline-rich domain (PD), DNA-binding domain (DBD), oligomerization domain (OD), and C-terminal domain (CTD). (**C**) The p53 transactivation domain (TAD) binds to MDM2, with the p53 TAD shown in green and MDM2 in yellow, as illustrated in PDB:1YCR [23]. (**D**) The illustration depicts the p53 tetramer binding to DNA via its DBD domain, with the DNA double helix represented in the center. The magenta, green, yellow and red colors represent the DBDs of individual p53 monomers, derived from PDB:3KMD [24]. (**E**) The illustration depicts the formation of a tetrameric complex comprising four p53 monomers, which interact through their oligomerization domains (OD). The ODs of the four monomers are represented by different colors: magenta, green, yellow and red, respectively. This illustration is based on the structure of p53 (PDB:2J0Z) [25].

*TP53* is located on chromosome 17p13.1 and encodes the 393-amino-acid p53 protein (Figure 1A). Structurally and functionally, p53 consists of six domains (Figure 1B) [19]: the transactivation domains (TAD1 and TAD2), which activate and enhance the transcription of target genes through interaction with cofactors (Figure 1C); the proline-rich domain (PD), which mediates nuclear export and stabilizes p53; the DNA-binding domain (DBD), which determines the DNA-binding characteristics of p53 (Figure 1D); the oligomerization domain (OD), which facilitates the formation of p53 tetramers, required for its full transcriptional activity (Figure 1E); and the C-terminal domain (CTD), which is involved in the regulation of p53’s activity and DNA-binding capacity [20]. Under stress stimuli, p53 monomers oligomerize across the OD to form a transcriptionally active tetramer that recognizes and binds specific DNA sequences [21,22] and regulates the transcription of downstream genes to control cellular biological processes. p53 also participates in cellular regulation through non-transcriptional functions, mediated by protein–protein interactions.

### 2.2. Regulation of p53 Expression

**Figure 2:**
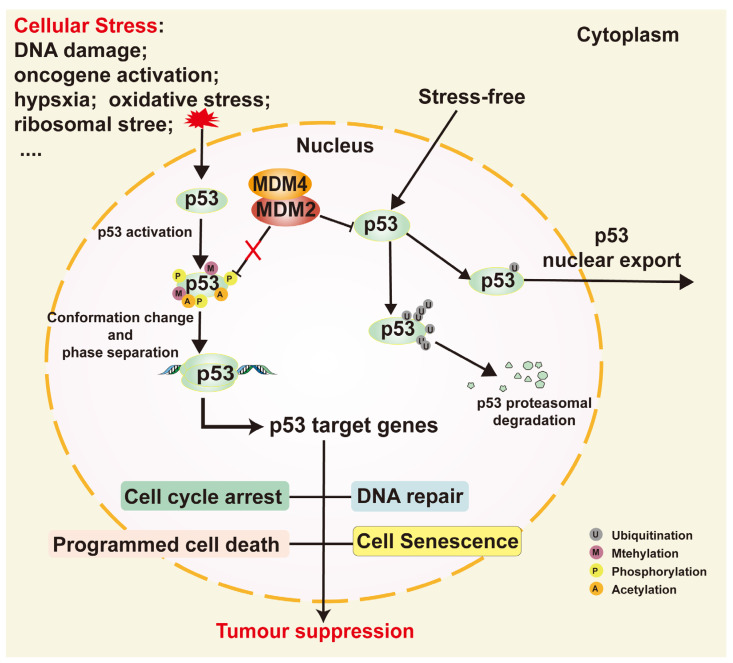
Regulation of p53 expression. (1) MDM2/MDM4 play a role in maintaining p53 homeostasis under non-stress conditions by regulating p53 ubiquitination. (2) MDM2/MDM4-mediated p53 monoubiquitination facilitates its nuclear export. The MDM2/MDM4-mediated polyubiquitination of p53 facilitates its proteasomal degradation. (3) The phosphorylation of p53 has been demonstrated to antagonize the interaction between MDM2/MDM4 and p53, thereby mediating the accumulation and activation of p53 protein levels. (4) The methylation and acetylation of p53 regulate its transcriptional activity and target specificity towards DNA.

Post-translational modifications (PTMs) are the principal mechanisms through which p53’s protein levels and functions are regulated (Figure 2) [9]. Under non-stress conditions, p53 is continuously expressed and maintained at low protein levels through proteasomal degradation pathways. Ubiquitination mediated by the Mouse Double Minute 2 (MDM2) homolog and Mouse Double Minute 4 (MDM4) homolog plays a central role in maintaining p53 homeostasis [26,27]. MDM2 and MDM4 are crucial negative regulators of p53. The N-terminal domain of MDM2 binds to the TAD1 of p53, mediating p53’s ubiquitination through the ubiquitin ligase activity of its RING domain. High MDM2 activity drives the polyubiquitination and subsequent proteasomal degradation of p53, whereas low MDM2 activity leads to monoubiquitination and the export of p53 from the nucleus [28]. MDM4, lacking E3 ubiquitin ligase activity, does not directly promote p53 ubiquitination. Instead, it binds to MDM2 via the RING domain, forming a heterodimer that prevents MDM2 homodimer formation and inhibits MDM2 self-ubiquitination [29]. The interaction of MDM2 and MDM4 with p53 is a crucial mechanism in maintaining p53 homeostasis.

In addition to ubiquitination, other PTMs, such as phosphorylation, acetylation and methylation, are also important mechanisms in regulating p53’s function. Phosphorylation and dephosphorylation significantly influence the activity and stability of p53, acting as essential regulatory switches within the p53 pathway. During cellular stress, most phosphorylation sites on p53 are phosphorylated, thereby activating the p53 pathway. This phosphorylation enhances the protein stability by reducing the affinity of p53 for MDM2/MDM4, which leads to the accumulation of p53 protein levels. Once the stress is resolved, dephosphorylation restores p53 to its homeostatic state [9,30]. However, certain phosphorylation sites, such as T55, S376 and S378, exhibit constitutive phosphorylation in unstressed cells, promoting p53’s degradation via the proteasomal pathway, while dephosphorylation during cellular stress enhances p53’s protein stability [9,31]. The acetylation and methylation of p53 have also been confirmed to play crucial roles in regulating p53’s function. For instance, under cellular stress, co-activators such as CBP/p300 and PCAF/hGcn5 facilitate the acetylation of the p53 CTD at the K370, K372, K373, K381, K382 and K386 sites, thereby preventing p53’s ubiquitination and enhancing its ability to bind to small DNA oligomers, while also promoting the recruitment of co-activators and HATs to the promoters of p53-responsive genes [9,32,33,34]. The methylation of p53 predominantly occurs on lysine and arginine residues, and, depending on the modification site and the number of methyl groups added, it can either activate or repress p53’s transcriptional activity. For instance, monomethylation at K382 and K370 suppresses p53’s transcriptional activity, whereas monomethylation at K372 and demethylation at K370 enhance p53’s transcriptional activity. Methylation at the R333, R335 and R337 sites influences the target specificity of p53 [9,35,36,37].

Moreover, other PTMs, such as sumoylation, neddylation, glycosylation, hydroxylation, lactylation and O-GlcNAcylation, also contribute to the regulation of p53 by influencing its stability, conformation and subcellular localization [8,9,38,39,40,41,42,43]. p53 serves as a central hub in coordinating multiple cellular responses to various stresses, playing distinct roles in different contexts [44,45]. The complex post-translational modifications of p53 influence its stability, conformation, localization and interactions with binding partners, either independently or through cooperative interactions [9,46,47,48,49]. These processes collectively contribute to the complexity of the p53 regulatory network.

## 3. Activation of p53 in Response to DNA Damage

DNA is continually subjected to damage from both internal and external factors, such as replication stress, telomere shortening, ultraviolet radiation and chemical toxins, among others [1]. If this DNA damage is not repaired in a timely manner, it can lead to genetic mutations and genomic instability, which in turn can trigger cancer. To mitigate the risks posed by DNA damage, cells have evolved sophisticated and highly coordinated defense mechanisms. These are largely mediated via a network of enzymatic cascades and signaling pathways that ensure genomic stability and accuracy. These mechanisms are referred to as the DDR [2]. The dysregulation or failure of DDR pathways is a major contributing factor to cancer initiation and progression, and it is closely linked to tumor heterogeneity and the development of drug resistance. The DDR network can be divided into three steps: (a) the detection of DNA damage, (b) the transmission of the signal and (c) the activation of effectors. The corresponding DDR-related molecules are classified as (a) DDR sensors, (b) DDR signal transducers and (c) DDR effectors [50]. The DDR kinases—ATR, ATM and DNA-dependent protein kinase (DNA-PK)—serve as critical sensors in this network. ATR responds broadly to most types of DNA damage, while ATM and DNA-PK specifically address double-stranded DNA breaks (DSBs). These kinases belong to the phosphatidylinositol 3-kinase-related kinase (PIKK) family, directly associating with DNA damage sites through DNA-binding cofactor complexes and being activated at these sites [51]. Subsequently, they phosphorylate the serine/threonine residues of their substrates (with a preference for SQ/TQ motifs), thereby activating downstream pathways, ultimately recruiting and activating effector proteins. These effector molecules regulate processes such as cell cycle progression, DNA repair, apoptosis, cellular metabolism and senescence.

**Figure 3:**
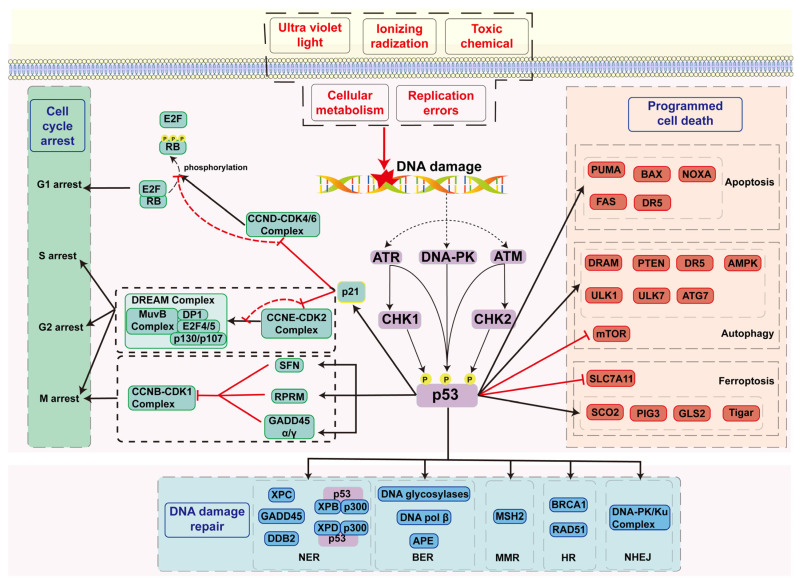
The roles of p53 in the DDR. (1) p53 plays a pivotal role in the DDR, functioning as the primary regulator of this process. (2) p53 plays a role in regulating the cell cycle through the transcriptional control of p21, SFN, RPRM and GADD45α/γ. (3) p53 is involved in the process of DNA damage repair, employing both transcription-dependent and transcription-independent mechanisms. (4) p53 plays a pivotal role in the initiation of programmed cell death (PCD), which serves as a mechanism for the elimination of damaged cells.

DNA damage is the first confirmed stressor for p53 [52]. p53 is a key substrate of DDR kinases and serves as a central effector in the DDR, playing an essential role in the entire process (Figure 3). p53 is activated by phosphorylation through CHK1 and CHK2, mediated by ATR and ATM, respectively. The activation of p53 is a critical component of the DDR, deeply involved in various biological processes of the DDR through both transcription-dependent and transcription-independent mechanisms [3,4]. First, p53 induces cell cycle arrest by upregulating the expression of the cyclin-dependent kinase inhibitor p21, thereby providing sufficient time for the recognition and repair of DNA damage. This prevents the transmission of erroneous DNA sequences to the replication phase and to the daughter cells. Second, p53 plays a significant role in various DNA damage repair pathways, regulating the activity of different DNA repair systems, such as homologous recombination (HR) and nucleotide excision repair (NER). Finally, when the repair mechanisms fail to effectively rectify the DNA damage, p53 activates apoptosis pathways to eliminate cells with potential mutations, thereby preventing tumorigenesis. Loss of function or mutations in p53 can result in the loss of this critical protective mechanism, significantly increasing the genomic instability and the risk of cancer development. Therefore, p53 plays an indispensable role in maintaining genomic stability. Notably, p53 can interact with various forms of damaged or altered DNA through its CTD, including deletion/insertion mismatches and DNA ends, as well as double-strand and single-strand breaks [53], and is capable of modulating the recognition patterns of its DBD [54]. Cells lacking wild-type p53 (wtp53) accumulate chromosomal abnormalities, such as amplifications, deletions, inversions and translocations.

## 4. Functional Roles of p53

### 4.1. Cell Cycle Arrest

Cell cycle arrest plays a crucial role in the DDR. This mechanism involves pausing the cell cycle to provide ample time for DNA repair, preventing cells with DNA damage from continuing to divide and passing on the damaged genome, thereby avoiding mutations and genomic instability. The regulation of the cell cycle is typically achieved through cell cycle checkpoint mechanisms [55]. These checkpoints can detect DNA damage and, through a series of signaling cascades, halt cyclin-dependent kinases’ (CDKs) activity, delay cell cycle progression or, in response to irreparable DNA damage, induce cell cycle exit or apoptosis [56]. The mechanisms of cell cycle arrest mainly involve CDKs and their regulatory factors, CDK inhibitors (CKIs), and cyclins [57,58]. The interaction between CDKs and cyclins is crucial for cell cycle progression, but, in the presence of DNA damage, these complexes are inhibited, preventing the cell from entering the next phase of the cycle. This mechanism relies on the activation of CHK1 and CHK2, thereby mediating CDK inactivation through the regulation of downstream signaling pathways. In this process, p53 and its target protein p21 (a crucial cell cycle inhibitor) play a key role (Figure 3) [59]. Of course, CHK1 and CHK2 can also trigger cell cycle arrest through p53-independent pathways, but the p53/p21 signaling pathway is undoubtedly the most important [60,61], and cell cycle arrest was one of the first functions proven for p53 [62]. Among the studies on the p53/p21 axis, the most extensively studied component is the retinoblastoma protein (RB). In response to DNA damage, activated p53 upregulates the expression of p21. As a CDKI, p21 inhibits CDK4/6–cyclin D and CDK2–cyclin E complexes, preventing the phosphorylation of the RB protein, maintaining the binding between RB and E2F and suppressing E2F’s activity. This blocks the progression of the cell cycle in response to DNA damage, preventing the cell from advancing to the next cycle [9,63]. Additionally, the p53/p21 axis can also mediate cell cycle arrest through the DP, RB-like, E2F and muti-vulval class B complex (DREAM complex). After activation, p53 upregulates the transcription of p21/CDKN1A, inhibiting CDKs, which leads to decreased phosphorylation levels of p107 and p130 and promotes the formation of the DREAM complex. The DREAM complex binds to E2F or CHR promoters to suppress their transcription, thereby inducing cell cycle arrest [28,64]. Furthermore, p53 can regulate the cell cycle by influencing the transcription of 14-3-3 Sigma (SFN), Reprimo (RPRM) and GADD45 [58,65,66,67]. Signaling kinases phosphorylate and activate p53, while stalling replication forks. p53, in turn, activates downstream target genes, which influences cell cycle arrest and promotes DNA repair to affect the progression of replication forks. Additionally, p53 plays a role in silencing Long Interspersed Nuclear Element-1 (LINE-1) by limiting its autonomous replication, further reducing replication stress [68]. Overall, p53 ensures the integrity and stability of replication forks by recruiting repair proteins, enhancing the stability, thereby playing a crucial role in cell cycle arrest.

### 4.2. DNA Damage Repair

p53 is a key player in DNA damage repair, and its ability to determine cells’ fates (survival or death) depends on the extent of the DNA damage [69]. To achieve this, p53 integrates signals from a range of DNA damage sensors and repair pathways. Such sensors, including ataxia telangiectasia mutated (ATM) and ataxia telangiectasia and Rad3-related (ATR), detect DNA damage and initiate signaling cascades that ultimately lead to the activation of p53. The degree of p53 activation is subject to stringent regulation, with the level of DNA damage serving as a key determinant [70]. For example, minor DNA lesions may result in a moderate increase in p53 activity, which in turn promotes cell cycle arrest and DNA repair. Conversely, extensive or irreparable DNA damage may result in elevated levels of p53 activation, which can then lead to either apoptosis or senescence [70]. In addition to its role in sensing DNA damage, p53 also regulates the expression of numerous genes involved in DNA repair, cell cycle arrest and apoptosis. The specific genes that are activated or repressed by p53 depend on the type and severity of the DNA damage, thereby further contributing to p53’s ability to tailor its response to the specific needs of the cell.

p53 plays a critical role in the DDR by regulating various downstream target genes, such as p21, SFN, RPRM and GADD45α/γ., and interacting with repair proteins like BRCA1 and DNA polymerase β, thereby enhancing the repair efficiency of non-homologous end joining (NHEJ) and HR (Figure 3). These mechanisms not only ensure cell survival in the face of DNA damage but also effectively prevent the accumulation of mutations, thereby reducing the risk of cancer. Studies have revealed that p53 has both transcription-dependent and transcription-independent functions in NER. As a transcription factor, p53 promotes the transcription of DDB2 (p48) and XPC in the early stages of NER. DDB2 forms a UV-DDB heterodimer with its binding partner, DDB1, which binds to 6-4 photoproducts (6-4PPs) and cyclobutane pyrimidine dimers (CPDs) to help recruit XPC, enhancing the cell’s ability to localize DNA damage and target repair [71]. Additionally, p53 exhibits transcription-independent activity in NER by regulating the helicase activity of XPB and XPD [72] and recruiting p300 histone acetylase to damage sites, where it acetylates histone H3 [73]. p53 modulates subunit chromatin accessibility through these two pathways.

### 4.3. Induction of Apoptosis

Under specific conditions, the cell death process strictly regulated by internal genetic programs is referred to as PCD, a concept first introduced by Lockshin in 1964 [74]. Currently, several important functions of PCD have been confirmed: sculpting structures and driving morphogenesis, deleting structures, regulating cell numbers and eliminating unwanted and potentially dangerous cells. Different PCD signaling pathways also exhibit cross-talk, with key players such as p53, mTOR and NF-κB playing significant roles in various forms of PCD [75]. In the context of the DDR, apoptosis serves as the final line of defense in eliminating abnormal cells and protecting genomic stability. p53 has been shown to play a pivotal role in apoptosis (Figure 3). For example, p53 promotes changes in mitochondrial membrane permeability by upregulating pro-apoptotic genes (e.g., BAX, NOXA and PUMA) and downregulating anti-apoptotic genes (such as BCL-2), leading to the release of cytochrome c and the activation of caspases, ultimately triggering apoptosis [76,77]. p53 also upregulates the expression of Fas and enhances the activity of the Fas-associated signaling pathway, increasing the sensitivity of cells to Fas-induced apoptosis [78]. Notably, p53 family members exhibit significant similarity to p53 in this apoptotic mechanism; for instance, BAX, PUMA and BCL-2 are common target genes [77]. p53 has the dual capability of promoting cell survival through DNA damage repair and inducing apoptosis, which may seem paradoxical. So, how does p53 determine the cell fate? The outcome of a cell following p53 activation is influenced by factors, such as the nature, duration and intensity of the stress signal, along with the specific context, like the cell type [44,45]. As for the choice between life and death in the DDR, the outcome is influenced by the severity of damage or the length of stress exposure; specifically, transient stress triggers a survival response (where damage is repaired), whereas intense stress with irreparable damage results in apoptosis or senescence (where abnormal cells are cleared) [70].

### 4.4. Senescence

Senescence can be triggered by various factors, including replicative senescence, DNA damage-induced senescence, oncogene-induced senescence and chemotherapy-induced senescence. Cellular senescence can be triggered throughout tumorigenesis, progression and even cancer treatment, underscoring its multifaceted roles in tumor suppression [79,80,81,82,83]. Despite the distinct types of cellular senescence, they share common characteristics: (a) the increased expression of β-galactosidase, which is a significant marker of senescence [84,85]; (b) the activation of signaling pathways such as p53/p21/Rb/mTOR, with p53 playing a critical role as a central regulator, where the extents of phosphorylation and accumulation of the protein are also considered important markers of cellular senescence [80,86,87,88,89,90]; (c) cellular responses involving the DDR, metabolic reprogramming and chromatin remodeling via the senescence-associated secretory phenotype (SASP), where the DDR occupies a central role by mediating permanent cell cycle arrest [86,87,91,92,93].

The role of p53 in cellular senescence is quite complex. First, the DDR plays a central role in cellular senescence, and p53 is the core regulatory factor of the DDR. The p53/p21/Rb signaling axis, triggered by the activation of p53, is regarded as the initiating step of cellular senescence through cell cycle arrest [94,95,96,97]. Furthermore, p53 regulates the NF-κB and mTOR signaling pathways and inhibits SASP, thereby alleviating the impact of senescent cells on the microenvironment and suppressing the dissemination of senescence signals [88,93,98,99,100,101]. Additionally, p53 inhibits senescence by inducing autophagy to eliminate damaged organelles and enhancing cellular resistance to oxidative stress [101,102]. p53 serves as a regulatory factor in cellular senescence. It maintains cellular homeostasis by regulating the cell cycle, inducing the DDR, modulating the SASP and interacting with other signaling pathways. p53 acts as a key trigger for senescence initiation in cellular senescence by regulating multiple pathways, such as cell cycle arrest, DNA damage repair, autophagy and inflammatory responses, and also exhibits anti-aging effects by limiting SASPs under certain conditions, with its function dependent on the specific cell type and stress context.

## 5. Abnormalities in p53 and Cancer Progression

### 5.1. Loss of Function (LOF) and Gain of Function (GOF): The Dual Impact of TP53 Mutations

**Figure 4:**
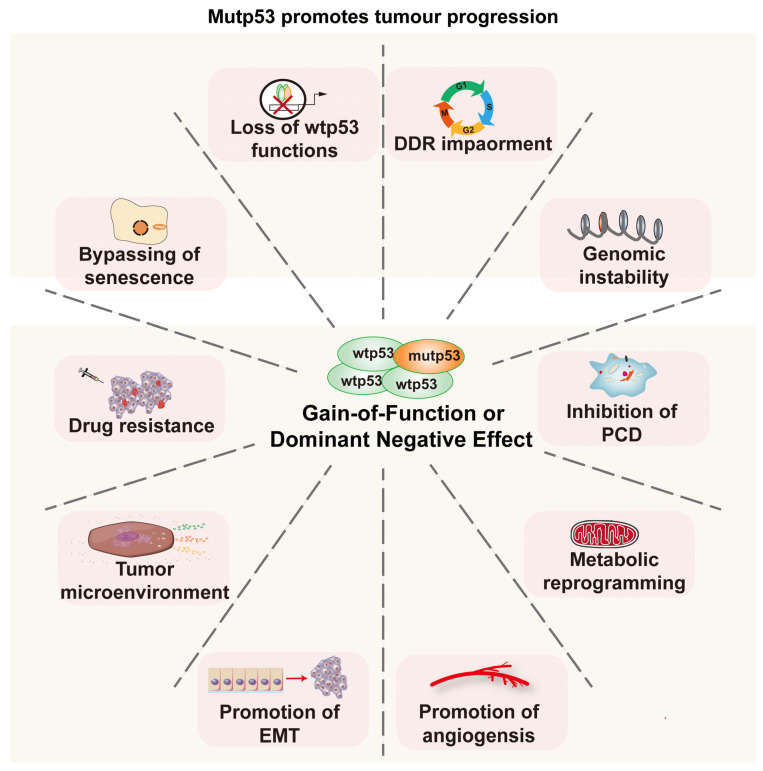
mutp53 promotes tumor progression. (1) mutp53 not only loses its normal tumor-suppressive function but also inhibits the function of wtp53 through a dominant negative effect, thereby leading to the failure of the DDR and genomic instability. (2) GOF mutp53 facilitates tumor progression through mechanisms including metabolic reprogramming, the modulation of the tumor microenvironment, the facilitation of tumor–stroma transition and the enhancement of angiogenesis.

*TP53* mutations have been identified in up to 50% of human tumors, with varying mutation frequencies across different cancer types. In some cancers, the mutation frequency can reach as high as 80%, making it the most commonly mutated gene [8,16,17]. Missense mutations are the primary type of *TP53* mutation found in tumors, and hundreds of *TP53* mutations have been identified. However, the majority of these occur within the p53 DBD. Six hotspot mutations—R175H, R248Q, R273H, R248W, R273C and R282W—account for 30% of all p53 mutations [103,104,105]. Missense mutations of p53 can be categorized into DNA-binding mutations and structural mutations. DNA-binding mutations involve the replacement of amino acid residues responsible for specific DNA binding, leading to altered regulatory functions over target genes. Structural mutations result from alterations in amino acids that compromise the thermal stability of p53, leading to protein misfolding [106]. Notably, these two types of mutations are not mutually exclusive; for instance, the R248Q mutation exhibits both DNA binding and structural mutation characteristics [107]. Mutant p53 (mutp53) is unable to adequately respond to cellular stress, particularly DNA damage. However, p53 is a pivotal factor in the entire DDR biological process. The failure of p53-mediated mechanisms allows cells to evade cell cycle checkpoints, repair DNA damage with low fidelity and avoid apoptosis, ultimately resulting in mutation accumulation and carcinogenesis (Figure 4). From an allele perspective, it seems that both alleles of p53 need to be mutated to cause a loss of function. However, as p53 functions as a tetramer, a single allele mutation can compromise its activity. This occurs as mutp53 disrupts the tetramer formation of wtp53 through hetero-oligomerization, effectively silencing p53’s function. This mechanism is referred to as the dominant negative effect or LOF (Figure 4) [108,109].

Mutations in p53 can have even more severe consequences as certain mutp53 proteins not only lose their original tumor suppressor functions but also acquire new functions that promote tumorigenesis, referred to as GOF mutations. mutp53 contributes to a tumor microenvironment that favors tumor progression, promotes immune suppression and evasion and potentially enhances tumors’ resistance to therapies (Figure 4) [106]. The oncogenic activity of GOF mutp53 is primarily mediated through the following four mechanisms: (1) the regulation of gene transcription via interactions with other transcription factors [110]; (2) specific DNA binding through the CTD domain [111,112]; (3) the mediation of chromatin remodeling [113]; (4) the acquisition of specific DNA-binding activity [114,115]. Furthermore, metabolites derived from the gut microbiota have been shown to facilitate the conversion of mutp53 into oncogenic factors, indicating its potential for environmentally dependent oncogenicity [116]. Additionally, mutp53 lacks the activity to initiate the transcription of MDM2/MDM4 and evade degradation by MDM2 through the protection of chaperone proteins such as HSP90, resulting in its accumulation within tumor cells and exacerbating the impact of mutp53 [106,117].

### 5.2. MDM2: Another Central Factor in p53 LOF

**Figure 5:**
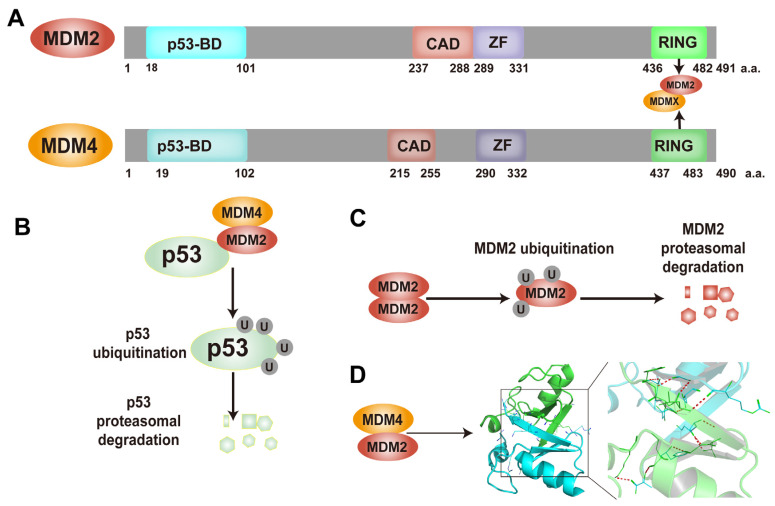
MDM2/MDM4 suppress p53. (**A**) Schematic representation of the MDM2/MDM4 protein structure [128]. p53-BD: p53-binding domain; CAD: central acidic domain; ZF: zinc finger domain; RING: C-terminal RING domain. (**B**) The MDM2/MDM4 complex facilitates the ubiquitination and subsequent proteasomal degradation of p53. (**C**) MDM2 forms homodimers through its RING domain and mediates its own ubiquitination and proteasomal degradation. (**D**) MDM2/MDM4 form heterodimers through their RING domains, which antagonize the formation of MDM2 homodimers. The structure is from PDB:2VJE [129].

MDM2 was discovered by George [118] and has been shown to be an oncogene [119]. As previously discussed regarding the regulation of p53’s expression levels, MDM2/MDM4 serves as the primary negative regulator of p53 by mediating its ubiquitination and proteasomal degradation, acting as a bridge by facilitating interactions between p53 and the components of the degradation machinery (Figure 5) [26,27]. MDM2/MDM4 amplification and the resulting low levels of the p53 protein have been identified in many tumors with wild-type p53. Additionally, research has found that the oncogenic activity of MDM2 extends beyond the regulation of p53, exhibiting non-p53-dependent oncogenic activity; for example, MDM2 can enhance the survival and proliferation of tumor cells by regulating serine metabolism [120]. Targeting MDM2 to restore proper p53 expression levels, thereby reinstating its tumor-suppressive function, represents a promising anti-cancer therapeutic strategy [121,122,123]. Currently, various MDM2 antagonists have been developed to stabilize the p53 protein and inhibit tumor progression by disrupting the protein–protein interaction between MDM2 and p53 or by targeting MDM2 for degradation.

In addition to MDM2 amplification, numerous additional factors contribute to the loss of p53 function in wild-type p53 tumors. For example, previous studies have demonstrated that p52-ZER6 facilitates p53 ubiquitination and proteasomal degradation by promoting and stabilizing the formation of the p53/MDM2 complex, thereby acting as a driving factor in cancer development [124]. Furthermore, CCDC106 competes with MDM2 and MDM4, directly interacting with the transactivation domain of p53, thereby downregulating the levels of both p53 and MDM2 and promoting cell proliferation and survival [125]. Furthermore, additional p53 inhibitors, including neurogenic differentiation factor 1 (NeuroD1) and pre-B-cell leukemia transcription factor 3 (PBX3), have been observed to inhibit the p53/p21 axis by binding to the p53 promoter and suppressing its transcriptional activity [126,127]. While these inhibitors have demonstrated potential in cancer therapy, it is evident that further in-depth research is required to fully realize their capabilities (Figure 5).

## 6. Abnormalities in p53 and DDR Provide Directions for Cancer Therapy

**Figure 6:**
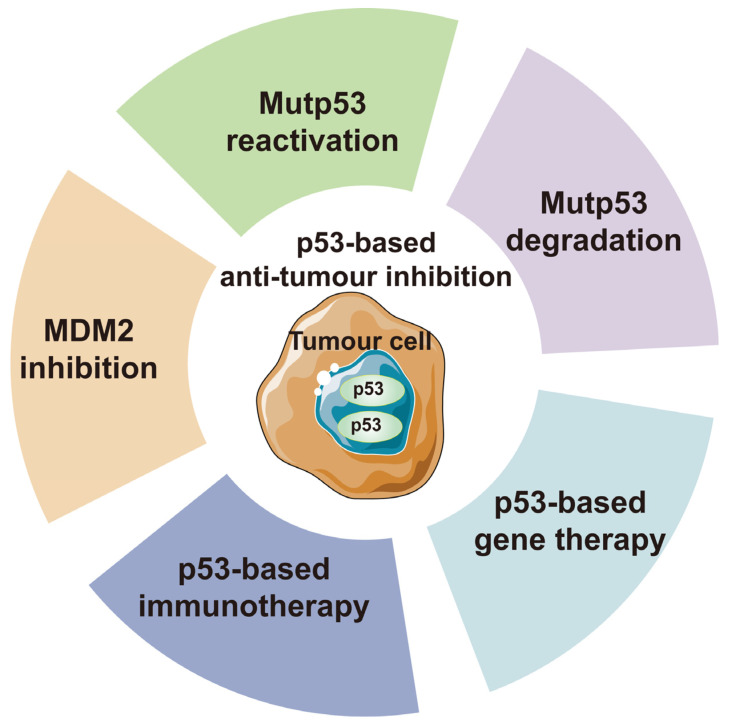
p53 based anti-tumor therapies. The current p53-based anti-cancer strategies can be classified into five categories. (1) restoring the function of mutp53; (2) degrading mutp53; (3) targeting negative regulators of p53; (4) p53-based immunotherapy; and (5) p53-based gene therapy. In the following sections, this article will review the p53-based anti-tumor therapeutic strategies that have entered clinical trial stages.

Given p53’s critical role in human cancer, overcoming the challenge of targeting p53 for anti-tumor therapeutic strategies could benefit the majority of cancer patients [130]. However, this goal has not been achieved due to the smooth surface of p53, lacking druggable pockets, and the extensive diversity of p53 protein mutations. Despite these challenges, p53 remains a promising anti-tumor therapeutic target due to its unique role. The current p53-based anti-tumor therapeutic strategies can be broadly categorized into two types (Figure 6). The first is aimed at restoring the tumor-suppressive functions that p53 is supposed to exert, including the reversion of mutant p53, antagonizing p53 negative regulators and overexpressing p53 through genetic approaches. The second involves other p53-derived anti-tumor approaches, such as p53-derived peptide-based immunotherapy and the degradation of mutp53. Based on these strategies, researchers have developed a variety of *TP53* or p53-based drugs, some of which have been approved for clinical trials. These studies provide hope for p53-based anti-tumor therapies.

### 6.1. Direct Targeting of p53

Restoring the normal function of p53 holds the potential to suppress cancer cell growth and enhance the efficacy of cancer therapies. These drugs exert their effects through two primary mechanisms: first, small-molecule compounds bind to mutp53 via a chaperone-like mechanism, restoring its wtp53 conformation and reactivating its tumor suppressor function; second, by promoting the degradation of mutp53, these compounds not only alleviate its inhibitory effect on wtp53 but also suppress the oncogenic activity of mutp53.

In 1999, the first compound with the capacity for mutp53 reactivation, CP31398, was reported. Identified by Pfizer by screening a synthetic compound library, CP31398 restored p53’s transcriptional activity and inhibited tumor growth in vivo. CP31398 features a Michael receptor (Michael addition) and restores the anti-tumor activity of p53 by stabilizing the active conformation of mutp53 and inhibiting its ubiquitination [131]. APR-246 is a methylated product of the small-molecule compound PRIMA-1, which is a prodrug. Its metabolite methylene quinuclidinone (MQ) covalently reacts with the thiol group of cysteine residues in the core domain of mutant p53 to restore its wild-type conformation, demonstrated to induce apoptosis in Saos-2 cells (p53^R273H^) and inhibit tumor formation in animal models [132]. Compared to PRIMA-1, APR-246 exhibits stronger anti-tumor activity, counteracting the enhancement of the inflammatory tumor microenvironment caused by mutant p53, restoring immune surveillance and inhibiting immune evasion [133,134]. COTI-2 is a novel thiosemicarbazone derivative identified through high-throughput computational screening. COTI-2 exhibits p53-dependent anti-tumor activity by binding to mutp53 and restoring its functional conformation. It also mediates p53-independent anti-tumor activity through AMPK activation and the inhibition of the mTOR pathway [135]. Phenethyl isothiocyanate (PEITC) is a naturally occurring dietary isothiocyanate, abundant in watercress and cruciferous vegetables, that exerts anti-tumor activity through various mechanisms, such as inhibiting tumor cell proliferation, inducing apoptosis and suppressing tumor invasion and metastasis [136,137,138]. PC14586 is a small molecule specifically designed to target p53Y220C, converting the Y220C-p53 mutant into the wild-type p53 conformation, thus activating the p53 transcriptional pathway and selectively inhibiting the proliferation of p53Y220C-mutant solid tumor cell lines [139]. As a drug with unique Chinese characteristics, historically regarded as a poison, arsenic trioxide, derived from this compound, has been used in the treatment of acute promyelocytic leukemia (APL), with promising results [140]. In 2021, Professor Min Lu and colleagues proposed that ATO exerts anti-tumor activity by restoring the wild-type conformation of mutp53. Through mass spectrometry and crystallographic analyses, they discovered that the arsenic atom in ATO can occupy a cavity formed by residues C124, M133, C135 and C141 within the p53 protein, thereby increasing the stability of p53 and restoring the tertiary structure and function of mutant p53 [141].

MCB613 is a small-molecule stimulant of steroid receptor co-activator (SRC), which was found by Padmanabhan et al. to selectively drive the rapid ubiquitination, nuclear export and lysosomal degradation of p53 R175H (GOF mutp53) through a USP15-dependent lysosomal pathway, with minimal effects on other mutants and wtp53 [142]. Currently, vorinostat (SAHA), romidepsin, panobinostat and belinostat have all entered clinical stages. Among these, SAHA exhibits preferential cytotoxicity against p53 mutant cells. Loss-of-function and gain-of-function experiments indicate that, while SAHA can induce various cellular effects, its cytotoxicity is largely driven by its strong capacity to destabilize mutp53 at the protein degradation level. The mechanism underlying this phenomenon involves the over-stabilization of mutp53 in tumors, as discussed previously, which is mediated by the upregulation of the chaperone protein HSP90. mutp53, by binding to HSP90, evades ubiquitination and proteasomal degradation mediated by the E3 ligases MDM2 and CHIP, leading to the stabilization of mutant p53. This chaperone activity of HSP90 is dependent on the deacetylation of its lysine 294 residue by HDAC6. SAHA inhibits HDAC6, thereby suppressing HSP90’s chaperone activity toward p53, which results in the release of mutant p53 from the stable mutp53–HSP90 complex. The released mutant p53 is then degraded via the MDM2 and CHIP E3 ligase pathways [143] (Table 1). Indeed, several drug candidates have successfully reactivated mutp53 in both in vitro and in vivo models. However, their clinical safety and efficacy still require additional validation. Challenges include the low efficiency of small-molecule reactivators, whether mutp53 regains its wild-type conformation with the help of these drugs and whether the effect is sustained or temporary, the unclear levels of p53 restoration required and the impact of tumor heterogeneity and resistance on efficacy. The absorption, distribution, metabolism and excretion (ADME) of these drugs must also be optimized to improve the therapeutic outcomes. Moreover, the low specificity of certain drugs may lead to off-target effects, potentially damaging normal cells, especially during radiotherapy and chemotherapy. Lastly, effectively activating p53 without causing damage to normal cells remains a critical challenge in the development of future therapeutic strategies [8,144,145].

### 6.2. Targeting the Negative Regulators of p53, MDM2 and MDM4

In tumor cells, the overexpression of MDM2 leads to the inhibition of p53 expression levels and activity, which is a key driving factor in tumorigenesis and progression. Therefore, antagonizing the ubiquitination of p53 by MDM2 or targeting the degradation of MDM2 is considered a promising anti-tumor strategy (Figure 6).

In 2004, Vassilev identified several MDM2 inhibitors through screening and structural optimization [41]. Among these, a series of cis-imidazoline derivatives known as Nutlins reactivated the p53 pathway by inhibiting MDM2. The core scaffold in Nutlins is cis-imidazoline, which structurally incorporates phenyl ring functional groups that resemble the three key residues of p53: Phe19, Trp23 and Leu26 This enables them to specifically interfere with the interaction between MDM2 and p53, promoting p53 protein accumulation and activation, thereby inducing apoptosis and exerting anti-tumor effects [41]. Currently, several derivatives from the Nutlin family have entered the clinical stages, including RO5045337 and Idasanutlin. AMG-232, also known as KRT-232, is an orally active and potent MDM2 inhibitor. It features a meta-chlorophenyl group, a para-chlorophenyl group and an isopropyl group that can mimic the three key residues of p53, occupying the binding site of MDM2 with p53 and blocking the interaction between MDM2 and p53, thereby activating the p53 signaling pathway. In vitro studies have confirmed that this molecule effectively activates p53 signaling and exhibits wild-type p53-dependent cellular activity. Tumor regression has been achieved in both in vitro experiments and in the MDM2-amplified SJSA-1 osteosarcoma model [152]. SAR405838 (MI-77301) specifically mimics the three key residues of wtp53 that bind to MDM2, and it interacts with the MDM2 drug-binding pocket, enhancing its affinity by inducing the refolding of the unstructured N-terminal region of MDM2. Notably, a single oral dose of 200 mg/kg of SAR405838 resulted in the complete regression of tumors in SJSA-1 xenograft mice [153]. APG-115 (AA-115) is a compound designed by Aguilar et al. to address the slow isomerization of SAR405838 in solution; it can rapidly and irreversibly convert into a single non-racemic isomer, exhibiting better oral pharmacokinetic properties and a higher IC50 value, effectively inhibiting the growth of wild-type p53 human cancer cell lines at low concentrations [154]. Milademetan is a dibenzodiazepine compound that competitively binds to MDM2, inhibiting the ubiquitination and nuclear export of p53. This action promotes the accumulation of p53 protein levels and activates the p53 pathway, inducing cell cycle arrest and apoptosis. It exerts p53-dependent anti-tumor activity both in vivo and in vitro [155]. NVP-CGM097 is a compound designed using computer-aided drug design techniques based on X-ray crystallography data, featuring a dihydroisoquinolinone core. It occupies the binding pocket of MDM2 at three critical residues, W23, L26 and F19, interrupting MDM2’s inhibitory effect on p53 and activating the p53 pathway [156]. HDM201 (Siremadlin) is a modified imidazopyrrolidinone analog based on the dihydroisoquinolinone core of NVP-CGM097, with a significantly stronger affinity for MDM2. HDM201 selectively inhibits MDM2, induces p53 expression, suppresses cell growth and induces apoptosis [157,158]. ASTX295 is a small-molecule MDM2 inhibitor developed by Astex Pharmaceuticals. It restores the tumor-suppressive activity of p53 by inhibiting the interaction between MDM2 and p53. Its molecular formula and mechanism of action have not yet been fully disclosed. KT-253 can inhibit the p53-dependent upregulation of MDM2 triggered by small-molecule MDM2 inhibitors, demonstrating improved efficacy, particularly in MDM2-overexpressing tumors with intact *TP53* [159].

Stapled peptides are a special class of peptides that enhance the structural stability by introducing chemical cross-links at specific positions, improving cell permeability and metabolic stability. This modification allows peptides to achieve better targeting, cell penetration and pharmacokinetic properties, making them important in drug development targeting protein–protein interactions. ALRN-6924 is a dual-specific stapled peptide designed and optimized from the α-helical sequence of the transactivation domain of p53, specifically targeting MDM2 and MDM4 to activate p53 signaling in wtp53 cells. ALRN-6924 exhibits good cell permeability, solubility, pharmacokinetics and safety. It is stable in ex vivo plasma but is hydrolyzed intracellularly to produce ALRN-8714. Although ALRN-8714 has stronger target affinity than its parent compound, it has lower permeability and is less effective when applied directly to cells than ALRN-6924 [160]. KT-253 is a protein degrader that targets MDM2, restoring the p53 protein levels and activity by degrading MDM2.

Targeting MDM2 and MDM4 to restore p53 expression and activity is a promising anti-cancer therapeutic strategy that has demonstrated efficacy in preclinical and clinical trials. However, no MDM2 inhibitor has yet been approved for clinical use. This is primarily due to several challenges faced by MDM2 inhibitors. (1) Most MDM2 inhibitors function by antagonizing the MDM2–p53 interaction, thereby preventing MDM2-mediated p53 degradation. However, this mechanism may lead to increased MDM2 protein levels, which could result in adverse effects. (2) These inhibitors show limited efficacy in tumors with mutp53, as they are primarily developed for wtp53 tumors and are less effective in tumors with p53 deletions or heterozygous mutations. (3) Some MDM2 inhibitors lack sufficient specificity, potentially leading to side effects and damage to normal cells, particularly in organs with high cellular turnover, such as the liver and bone marrow. To address these challenges, there is an urgent need for the development of more selective agents. Overcoming these challenges necessitates further investigation into the specificity and efficacy of MDM2 inhibitors, as well as the exploration of combination therapies [41,145,161,162] (Table 2).

### 6.3. Gene Therapy and Immunotherapy Based on p53

In the 1990s, two types of p53-based gene therapies entered clinical trials. One involved the introduction of the wtp53 gene into tumors using modified adenoviruses to activate the p53 pathway, while the other utilized modified adenoviruses to selectively infect and kill cancer cells (oncolytic viruses). However, due to severe adverse reactions observed in clinical trials, p53-based gene therapies were nearly discontinued [12]. With the completion of the Human Genome Project, advancements in gene editing techniques and the development of delivery vectors, gene therapy has once again become a popular topic in gene treatment. Currently, p53-based gene therapies primarily focus on reactivating the p53 pathway by introducing normal p53 genes into tumor cells (Figure 6). Depending on the delivery method, gene therapy can be categorized into viral and non-viral methods. The viral approach uses retroviruses as vectors to deliver genes into host cells, such as Gendicine (rAd-p53) [171], while the non-viral approach involves targeted delivery for transfection, such as SGT-53 [172]. These can be further divided into wtp53 DNA methods and wtp53 mRNA methods based on the different types of packaging materials. The former involves delivering wtp53 cDNA into target cells for expression, while the latter delivers wtp53 mRNA [173]. Additionally, there are other p53-based gene therapies, such as RNA interference, including Conseren [174]. The main challenges currently faced by p53-based gene therapy are the accuracy of gene delivery and the efficiency and safety of the delivery vectors.

p53-based immunotherapy has gained significant attention as a cancer treatment strategy in recent years (Figure 6). This approach enhances the immune system’s ability to recognize and eliminate tumor cells with abnormal p53 protein expression. This is achieved through the presentation of p53-derived peptides on the cell surface via major histocompatibility complex (MHC) molecules. The overexpression of mutp53 in tumor cells increases the presentation of p53-derived peptides. However, this strategy currently faces two challenges: firstly, some tumor cells exhibit very low proteasomal efficiency for mutp53, which hinders the presentation of p53-derived peptides; secondly, certain rapidly dividing normal cells express high levels of p53, which may lead to their recognition and attack by the immune system. Nevertheless, this approach is still regarded as a promising cancer treatment strategy. Currently, several anti-tumor therapies based on this mechanism have been developed, including the SLP-p53 vaccine, MVAp53 vaccine, DC-p53 vaccine and anti-p53 TCR therapy [175,176,177].

## 7. Discussion

Loss of p53 function or mutation is a hallmark of many cancers, underscoring its critical role in tumor suppression. Mutant p53 not only loses its tumor-suppressive function, but may also acquire pro-oncogenic properties, thereby promoting cancer progression, increasing treatment resistance and leading to poor patient outcomes. The diversity of p53 mutations results in heterogeneity in the tumor behavior and treatment response, presenting challenges and opportunities for cancer management. In recent years, there has been mounting evidence to suggest that mutant p53 proteins are capable of engaging in GOF activities that are distinct from the LOF phenotypes typically associated with these mutations. These GOF activities can include novel transactivation capabilities, altered protein interactions and changes in subcellular localization, all of which can contribute to cancer progression [178,179]. For example, some mutant p53 proteins have been demonstrated to interact with other transcriptional regulators and co-factors, thereby activating gene expression programs that support tumor growth and survival [180,181]. In certain instances, these interactions have been observed to occur in a sequence-specific manner, albeit distinct from the binding sites of wild-type p53. Consequently, while mutant p53 may lack the capacity to transactivate wild-type p53 target genes, it may acquire the ability to activate entirely new sets of genes. It is crucial to emphasize that the acquisition of mutant p53-specific transactivation activity is not a universal phenomenon. Furthermore, the precise mechanisms underlying these GOF activities remain under active investigation and are not yet fully understood.

Recent findings suggest great potential for therapeutic benefits by targeting the p53 pathway, presenting therapeutic potential. Strategies aimed at restoring wild-type p53 function, inhibiting mutant p53-enhanced activity or modulating its upstream and downstream effectors are being explored. Moreover, understanding the interplay between p53 and other signaling pathways in the tumor microenvironment has opened new avenues for the development of combination therapies to overcome resistance mechanisms. Hence, uncovering the complexity of p53’s function remains an important research topic in cancer biology and therapy. Future research will focus on the structural dynamics of p53, its interactions with other cellular proteins and the impact of associated mutations, which will enhance our ability to develop effective cancer prevention and treatment strategies [8,12,13,106]. Its complex role in the DDR and tumor suppression demonstrates the sophisticated mechanisms evolved by cells to maintain genomic stability and prevent malignant transformation. Therefore, future research needs to focus on deciphering the regulation of p53 activity, exploring the relationships between p53 and other signaling pathways in the DDR and evaluating the potential of p53 targeting for cancer treatment in the context of personalized medicine [50,182]. Meanwhile, elucidating the role of p53 in emerging fields such as immune regulation and metabolic regulation will further deepen our understanding of its multifaceted role in cancer and may reveal new treatment strategies. Therefore, it can be said that, in the fight against cancer, continued efforts to uncover the complexity of p53 are expected to lead to major breakthroughs.

## 8. Conclusions

In the complex field of the DDR and cancer progression, p53, as the ‘guardian of the genome’, plays a vital role in the network of cell homeostasis [183]. p53 is involved in multiple cellular processes, including detecting DNA damage, initiating appropriate repair mechanisms and triggering cell death when repair is not possible, which is essential in maintaining genomic integrity and preventing cancer progression. p53 coordinates a series of events, such as cell cycle arrest, DNA repair, apoptosis and senescence, by participating in the complex process of the DDR, thereby ensuring that potentially cancerous cells are effectively eliminated [7,8]. When DNA is damaged, p53 is stabilized and activated through complex mechanisms such as post-translational modifications and protein–protein interactions, allowing the cell to mount a tailored response to various stress signals. This review elucidates the complex interplay between p53 and the different DDR pathways and highlights its central and essential role in detecting DNA damage, signaling and executing appropriate cellular responses. The dual nature of p53 as a tumor suppressor and a key player in the DDR further highlights its importance in cancer biology. A deeper understanding of the mechanisms underlying p53’s regulatory functions and its interactions with other molecular players will help to uncover the mechanisms of cancer pathogenesis and provide potential paths for therapeutic intervention.

## Figures and Tables

**Table 1:** Direct targeting of p53.

Drug	Molecular Mechanism	Chemical Formula	Reported By (or Associated with p53)
APR-246	Restoring p53^R273H^ conformation induces mitochondrial pathway apoptosis	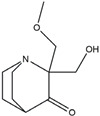	Ali et al., 2008 [146]
COTI-2	Restoring the DNA-binding characteristics of mutp53	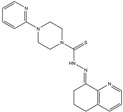	Salim et al., 2016 [147]
PEITC	Restoring the conformation of p53R175H and p53P223L blocks the TGF-β1/Smad pathway and activates the MPK pathway	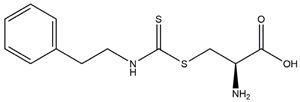	Matzinger et al., 1995 [148]
ATO	Enhancing the stability of the p53 protein restores the wild-type conformation of mutp53	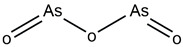	Chen et al., 2021 [141]
PC14586	Restoring the conformation of p53^Y220C^	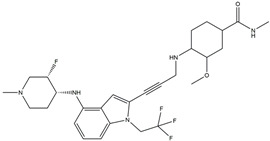	Dumble et al., 2021 [149]
MCB613	Drives rapid ubiquitination, nuclear export and lysosomal degradation of p53^R175H^	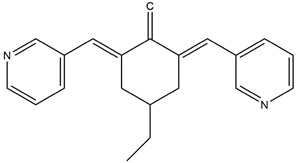	Wang et al., 2015 [150]
Vorinostat	Inhibits HDAC6, a key positive regulator of HSP90; disrupts the HDAC6–Hsp90 axis; and reduces the stability of mutp53	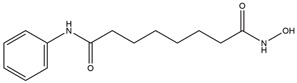	Vrana et al. 1999 [151]

**Table 2:** Targeting the negative regulators of p53, MDM2 and MDM4.

Drug	Molecular Mechanism	Chemical Formula	Reported By
RO5045337 (RG7112)	MDM2/p53 antagonists	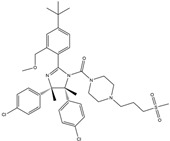	Andreeff et al., 2010 [163]
RO6839921 (RG 7775)	MDM2/p53 antagonists	Unpublished	Millard et al., 2011 [164]
Idasanutlin (RG7388)	MDM2/p53 antagonists	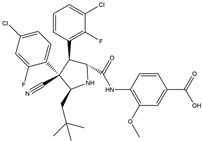	Ding et al., 2013 [165]
AMG-232 (KRT-232)	MDM2/p53 antagonists	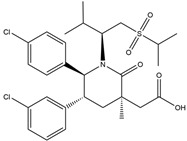	Sun et al., 2014 [166]
SAR405838 (MI-77301)	MDM2/p53 antagonists	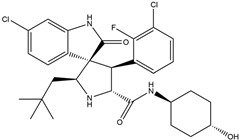	Wang et al., 2014 [153]
APG-115	MDM2/p53 antagonists	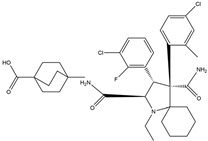	Aguilar et al., 2017 [154]
Milademetan	MDM2/p53 antagonists	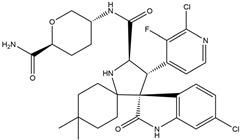	Andreeff et al., 2021 [167]
NVP-CGM097	MDM2/p53 antagonists	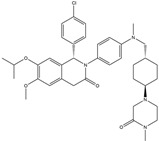	Holzer et al., 2015 [156]
HDM201 (Siremadlin)	MDM2/p53 antagonists	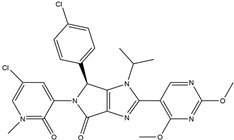	Furet et al., 2016 [157]
ASTX295	MDM2/p53 antagonists	Unpublished	Ahn et al. 2024 [168]
KT-253	Stapled peptides, MDM2/p53 antagonists	Unpublished	Schalm et al., 2023 [169]
Sulanemadlin (ALRN-6924)	MDM2/p53 antagonists, MDM4/p53 antagonists	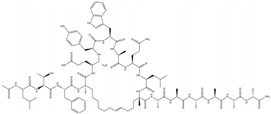	Meric-Bernstam et al., 2017 [170]

## Abbreviations

MDM2	Mouse Double Minute 2 homolog
MDM4	Mouse Double Minute 4 homolog
TAD	Transactivation domain
PD	Proline-rich domain
DBD	DNA-binding domain
OD	Oligomerization domain
CTD	C-terminal domain
RING	Ring finger domain
DDR	DNA damage response
PTMs	Post-translational modifications
DSB	Double-strand break
SSB	Single-strand break
BER	Base excision repair
NER	Nucleotide excision repair
MMR	Methyl-directed mismatch repair
HR	Homologous recombination
NHEJ	Non-homologous end joining
DREAM Complex	DP, RB-like, E2F and MuvB complex
MDC1	Mediator of DNA damage checkpoint 1
CTD	C-terminal domain
TCR	Transcription-coupled repair
GG-NER	Global genome repair
HOGG1	Human 8-oxoguanine glycosylase
PCD	Programmed cell death
DBD	DNA-binding domain
mutp53	Mutant p53
wtp53	Wild-type p53
GOF	Gain of function
LOF	Loss of function
SASP	Senescence-associated secretory phenotype
